# Supplementary material for: Linnemannia elongata (Mortierellaceae) stimulates Arabidopsis thaliana aerial growth and responses to auxin, ethylene, and reactive oxygen species
Source: PLoS One. 2022 Apr 12;17(4):e0261908. doi: 10.1371/journal.pone.0261908 (PMC9004744; doi:10.1371/journal.pone.0261908)
Supplement: S1 File — (DOCX) [file pone.0261908.s001.docx]

# Supplementary Materials and Methods

## Seed counting by automated image analysis in ImageJ

Preliminary manual image analysis to determine parameters:

1. Open sample image in ImageJ
2. Select Working Area, i.e. the area to analyze for that sample
3. Edit>clear outside
4. Image>Adjust>Threshold (unselect dark background, we used 1.25%)
5. Analyze> Analyze Particles
6. Summary, Outlines, Min = 10, Max = 1000
7. Show Results

Recording Macros for Batch Analysis

1. Start with an open image file, go to Plugins>Macros>Record
2. Take the image analysis steps determined in preliminary manual analysis
3. Hit “Create” and save in the ImageJ Macros folder

Using Macros for Batch Analysis

1. Process>Batch>Macro
2. Input & Output must be in SEPARATE FOLDERS to avoid overwriting input images
3. Open+ Point to macro in ImageJ Macro Folder
4. Process

In the case of the seed sheets analyzed in this study, there were four Working Areas in each raw image, one for each sample. This necessitated selecting each area in the raw image and exporting it as a separate input image to enable batch processing.

## CTAB-based DNA extraction protocol

Fungal mycelium was placed into 450 µL of 2x CTAB buffer (100 mM Tris-HCl 8.0 pH, 1.4 M NaCl, 20 mM EDTA, 2% CTAB, 4% PVP MW=10,000) and homogenized with a tube pestle. Next, 450 ul of 24:1 chloroform:isoamyl alcohol was added. The tubes were shaken briefly by hand, then centrifuged at 18,213 g for 8 min. The supernatant was removed and placed into a new tube. Chilled 2-propanol was added to the supernatant at 0.6 times its volume. The tubes were inverted about 20 times, then placed at -80°C for 8 minutes. The tubes were immediately placed in a pre-cooled 4°C centrifuge and centrifuged at 18,213 g for 15 minutes to pellet genomic DNA. The supernatant was discarded and the pellet washed with 800ul of chilled 80% ethanol, centrifuged at room temperature for 90s. This rinse was repeated exactly. All ethanol was aspirated from the pellet and the pellet vacuum dried for 2 minutes. The pellet was resuspended in 55ul of nuclease-free water. DNA concentration was quantified by absorbance using the Denovix DS-11 FXl.
