# Supplementary figures and images for: Linnemannia elongata (Mortierellaceae) stimulates Arabidopsis thaliana aerial growth and responses to auxin, ethylene, and reactive oxygen species
Source: PLoS One. 2022 Apr 12;17(4):e0261908. doi: 10.1371/journal.pone.0261908 (PMC9004744; doi:10.1371/journal.pone.0261908)

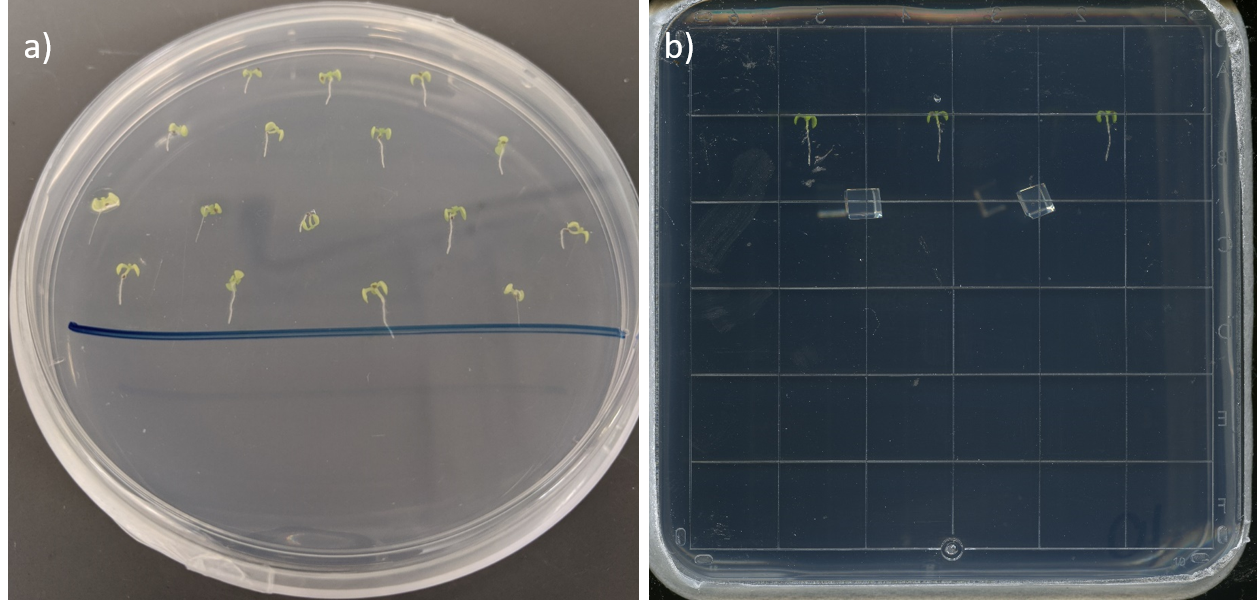

Supplement: S1 Fig — Panel a) 10 day old Arabidopsis thaliana seedlings on 1xMS germination plates and b) 11 day old Arabidopsis seedlings and blocks of media (colonized by fungi in fungal treatments or sterile in uninoculated control treatments) as arranged on PNM plates for the agar-based plant-fungal interaction experiments. (TIF) [file pone.0261908.s002.tif]

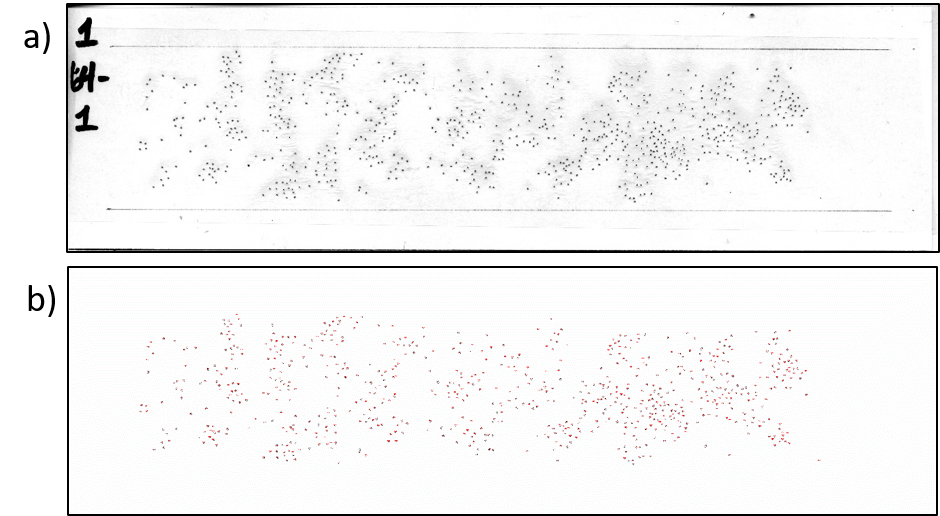

Supplement: S2 Fig — Arabidopsis thaliana was grown to maturity and the seeds of each plant collected by Aracon tubes and stored in Eppendorf tubes. After careful cleaning of the seed to remove stems, petals, and other plant debris, approximately 14 mg of seeds per sample were weighed on an ultrasensitive balance, adhered to a piece of white paper using a glue stick, covered by clear packing tape, scanned, and counted by image analysis in ImageJ. a) The scanned image of the subsampled seeds. b) The image analysis output, with areas identified as a “seed” outlined in red. (TIF) [file pone.0261908.s003.tif]

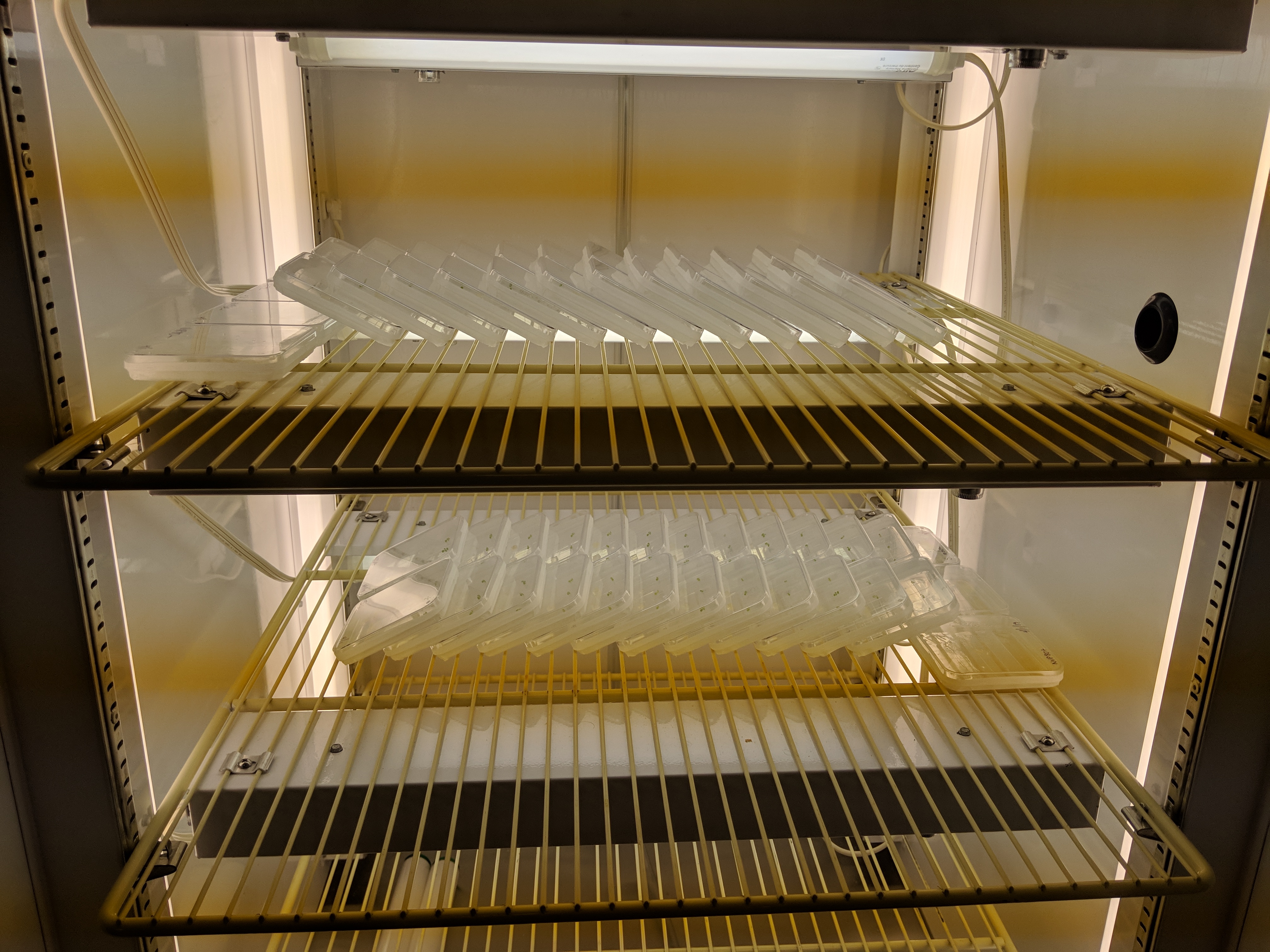

Supplement: S3 Fig — Arabidopsis thaliana seeds germination and Arabidopsis-L. elongata interaction studies were conducted on agar plates. These were incubated in a Percival growth chamber. Plates were stacked on a gentle angle to encourage smooth directional root growth along the agar surface. (JPG) [file pone.0261908.s004.jpg]

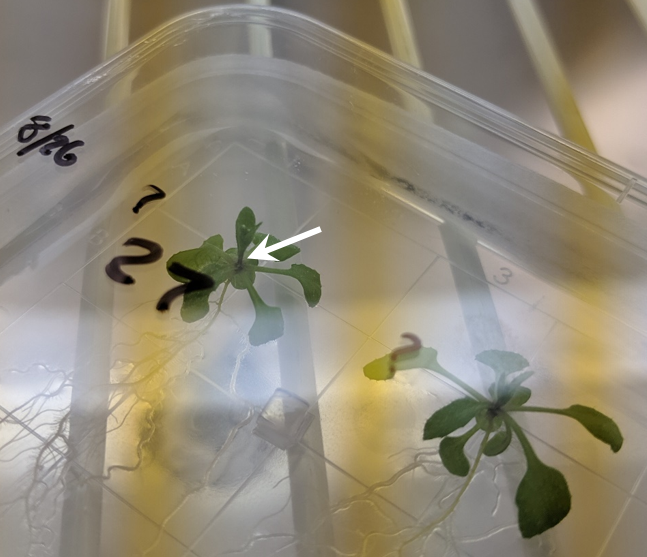

Supplement: S4 Fig — The arrow indicates the elongation of the Arabidopsis thaliana inflorescence away from the rosette of leaves which was considered to indicate “bolting.” (TIF) [file pone.0261908.s005.tif]

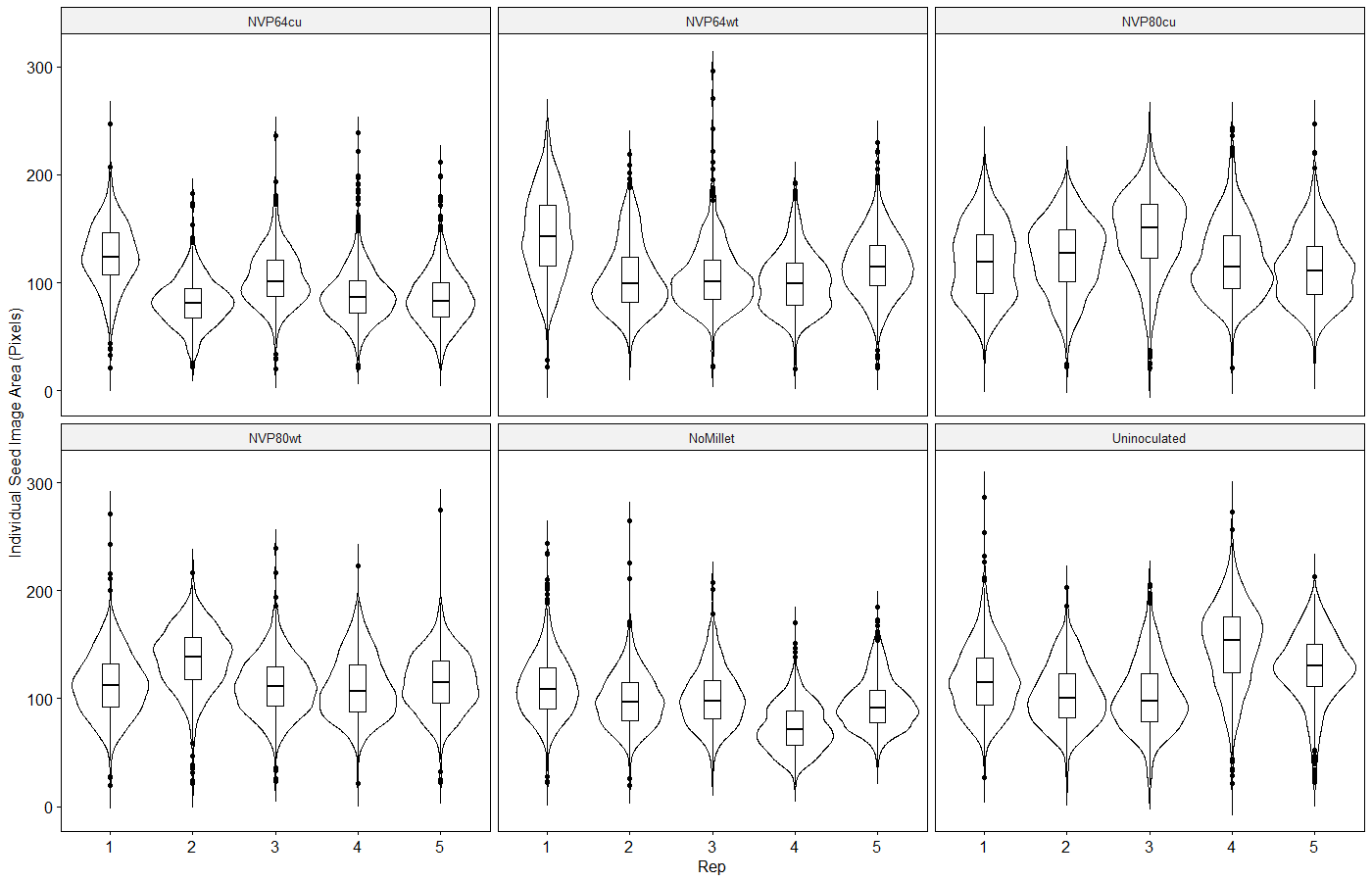

Supplement: S5 Fig — Arabidopsis thaliana was grown to maturity and the seeds collected by Aracon tubes. Facet names indicate the composition of the potting mix in which Arabidopsis plants were grown. The untreated control (NoMillet) contrasted treatments where the sterile potting soil was mixed 97:3 v:v with sterile millet mix (Uninoculated), or millet mix inoculated with one of four Linnemannia elongata strains (NVP64cu, NVP64wt, NVP80cu, or NVP80wt). A subset of seeds from 5 samples per treatment (sample indicated by ‘Rep’) were adhered to white paper and imaged using an Epson scanner. The y-axis indicates the pixel count of each individual seed scanned for each rep and treatment using ImageJ. (TIFF) [file pone.0261908.s006.tiff]

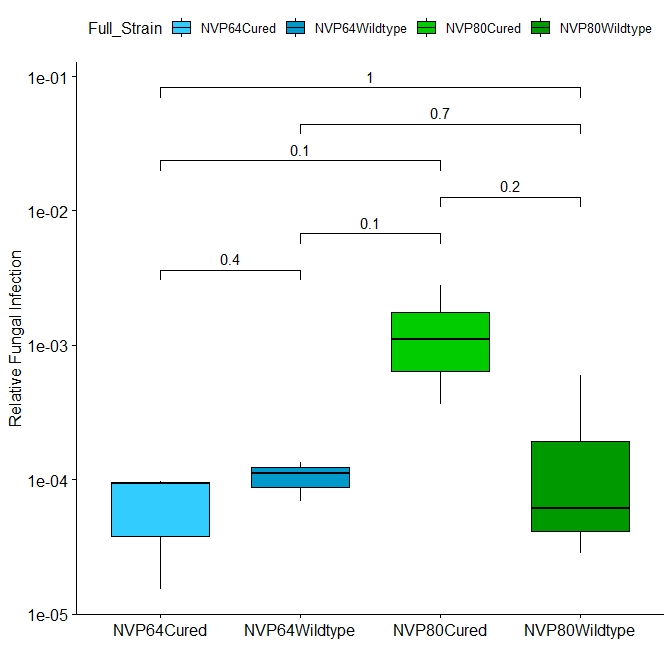

Supplement: S6 Fig — RNA was extracted from Arabidopsis thaliana roots colonized by Linnemannia elongata, pooled from all three plants on each agar plate, from three plates per treatment. The inferred ratio of fungal:plant cDNA is based on the qPCR results and standard curves for each qPCR primer set. Since Arabidopsis GADPH and L. elongata RPB1 are single copy genes, the ratio of fungal and plant template provides a normalized estimate of fungal colonization of plant roots. (JPEG) [file pone.0261908.s007.jpeg]

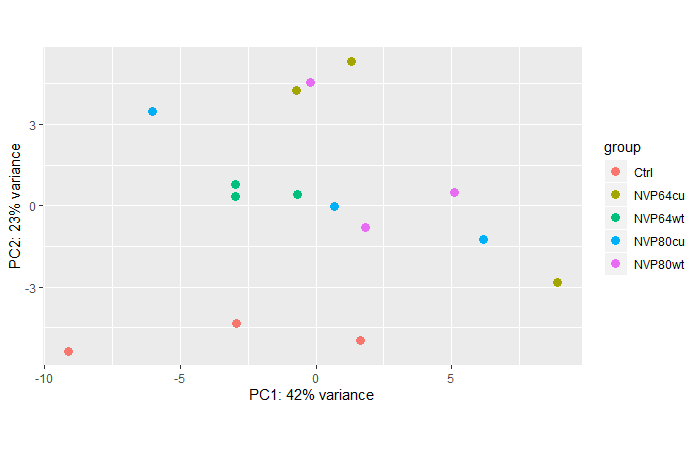

Supplement: S7 Fig — Arabidopsis thaliana root RNAseq data analyzed using DESeq2, sequenced from three biological replicates taken from each of the uninoculated control and fungal treatments inoculated with Linnemannia elongata. (TIFF) [file pone.0261908.s008.tiff]

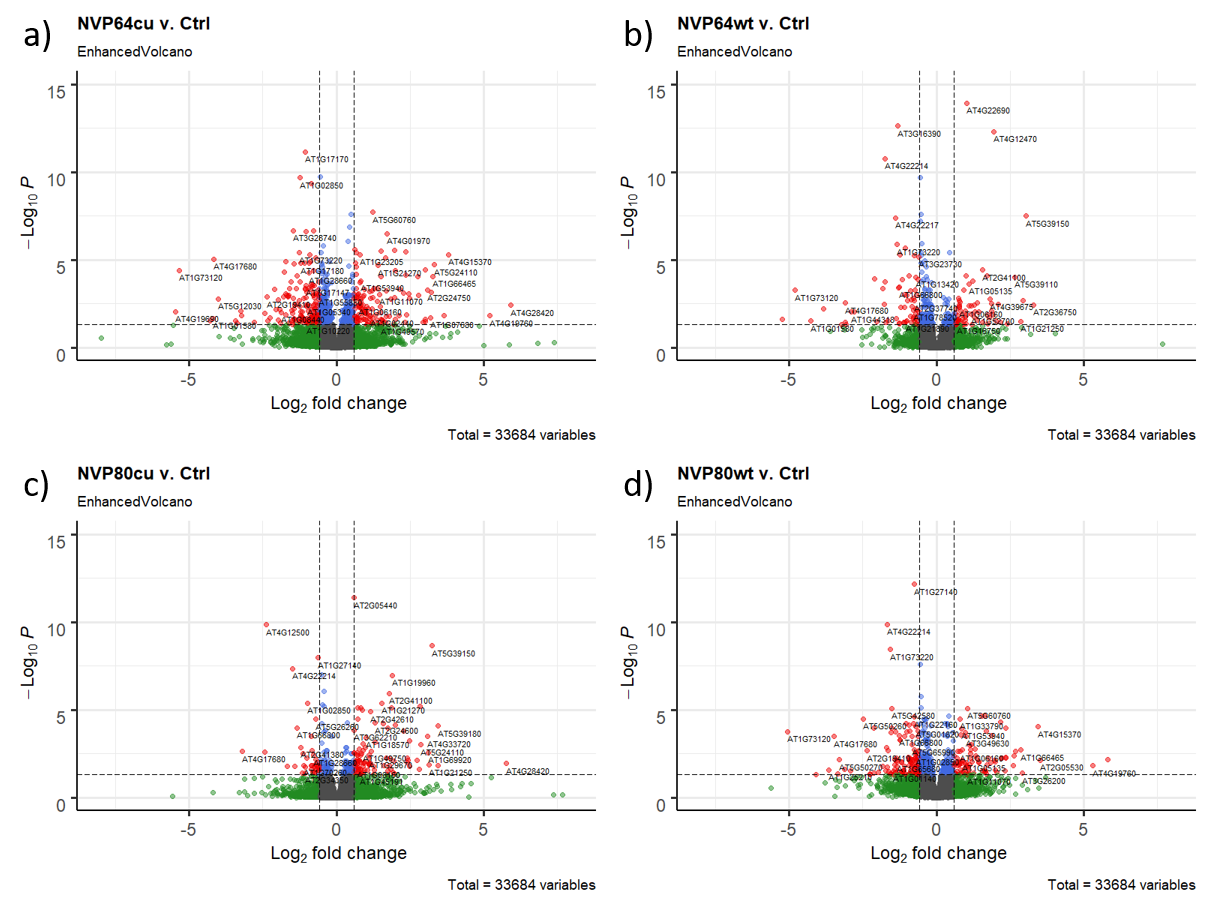

Supplement: S8 Fig — Pairwise comparisons of normalized Arabidopsis thaliana gene expression between fungal treatments and the uninoculated control, calculated from the DESeq2 analyses. Each point represents a gene, plotted by adjusted p-value and Log2 Fold Change (LFC) in expression between the fungal treatment and the control. Vertical dashed lines indicate the |LFC| = 1 threshold and horizontal lines indicate the adjusted p-value threshold of 0.05 used to identify genes with significant changes in expression. Genes are colored by which of the LFC and p-value cutoffs were exceeded: gray = failed both; green = exceeded only the LFC cutoff, but not the p-value cutoff; blue = exceeded p-value cutoff, but not LFC; red = exceeded both cutoffs. (TIF) [file pone.0261908.s009.tif]
