# Supplementary material for: Linnemannia elongata (Mortierellaceae) stimulates Arabidopsis thaliana aerial growth and responses to auxin, ethylene, and reactive oxygen species
Source: PLoS One. 2022 Apr 12;17(4):e0261908. doi: 10.1371/journal.pone.0261908 (PMC9004744; doi:10.1371/journal.pone.0261908)
Supplement: Attachment — Submitted filename: Recommendation.docx [file pone.0261908.s018.docx]

This is an interesting study to examine the role of *Linnemannia elongata* (Mortierellaceae) and its endosymbionts in Arabidopsis thaliana aerial growth, although endosymbionts had no significant effects on Arabidopsis performance and the expression of genes in Arabidopsis in this study. Overall, the study is solid, and the manuscript is well written. However, there are a few areas of the manuscript that could be strengthened a little.

Abstract: it may be necessary to state the significance of the study at the end of abstract.

Introduction:

L47: Please add the refs.

M&M:

Please provide some information about two isolates of L. elongata, NVP64 and NVP80. For example, where did you isolate?

L143. Please correct the format of the ref.

L193. Please show the ingredients for the ratio.

L234. How many replicates/plates for each treatment in the agar-based experiment.

L324. What do you mean of “2x”?

L352. Superscript

L373. Please correct the format of ref.

L.376. You randomly selected five plants for extracting root RNA. Why are there only three biological replicates?

Results:

L408. Somehow, fungal treatment can offset the negative effect of millet on plant growth?

L414. Fig. 3a

L416-417. From Fig. 3b, it seems that uninoculated control also had slightly higher average seed mass than NVP64wt and NVP80cu.

L428. Plants?

L433. Please correct the format of ref.

L443-451. It is not clear where are these data from. Please cite figures or tables here.

L485. How did you get this? Try to use statistical analysis (e.g., PERMANOVA) to reveal it.

L508-509. Please show the specific GOs here and site Table 2.

Discussion:

L529. It is surprising that bacterial endosymbionts negatively impact the growth of Linnemannia. According to the previous study (Büttner et al., 2021), bacterial endosymbionts (*Ca. Mycoavidus* necroximicus) can protect Mortierella/ Linnemannia from the attack of micro-predators.

L540-544. This sentence is too long. Try to break it down into two sentences.

L555. This sentence is less connected to previous ones. Is Serendipita (Piriformospora) indica phylogenetically close to *L. elongate*?

L637. Refs?

L649. Although K, P, and Fe are available in PNM medium, there is a great demand for plant growth in the fungal treatment. This is probably the main reason for so many down-regulated genes related to nutrients.

L651. Correct the format of ref.
