# Supplementary material for: Linnemannia elongata (Mortierellaceae) stimulates Arabidopsis thaliana aerial growth and responses to auxin, ethylene, and reactive oxygen species
Source: PLoS One. 2022 Apr 12;17(4):e0261908. doi: 10.1371/journal.pone.0261908 (PMC9004744; doi:10.1371/journal.pone.0261908)
Supplement: Attachment — Submitted filename: ResponseToReviewers.docx [file pone.0261908.s019.docx]

**We appreciate the reviewers for their time and comments. Below we provide a point-by-point response to the peer-reviews.**

**Reviewer 1**

This is an interesting study to examine the role of *Linnemannia elongata* (Mortierellaceae) and its endosymbionts in Arabidopsis thaliana aerial growth, although endosymbionts had no significant effects on Arabidopsis performance and the expression of genes in Arabidopsis in this study. Overall, the study is solid, and the manuscript is well written. However, there are a few areas of the manuscript that could be strengthened a little.

1. **Abstract: it may be necessary to state the significance of the study at the end of abstract.**

We’ve added a concluding significance statement:

“Together, these results indicate that beneficial plant growth promotion and seed mass impacts of *L. elongata* on Arabidopsis are likely driven by plant hormone and defense transcription responses after plant-fungal contact, and that plant phenotypic and transcriptional responses are independent of whether the fungal symbiont is colonized by *Mollicutes* or *Burkholderia*-related endohyphal bacteria.”

2. **Introduction : L47: Please add the refs.**

New references have been included.

3. **M&M: Please provide some information about two isolates of L. elongata, NVP64 and NVP80. For example, where did you isolate?**

This has been included

4. **L143. Please correct the format of the ref.**

This has been done.

5. **L193. Please show the ingredients for the ratio.**

This has been added: “transplanted into 4 in^3^ pots with SureMix mixed with the appropriate millet mix treatment at 3% by volume.”

6. **L234. How many replicates/plates for each treatment in the agar-based experiment.**

This has been added “The number of biological replicates per treatment varied by experiment as follows: 27 for the bolting assay, 12 for the media panel, and 27 for the endobacteria panel.”

7. **L324. What do you mean of “2x”?**

We replaced with ‘twice’

8. **L352. Superscript**

Fixed

9. **L373. Please correct the format of ref.**

Fixed

10. **L.376. You randomly selected five plants for extracting root RNA. Why are there only three biological replicates?**

This was added to at LN311 to clarify: ‘three of which were selected as triplicate biological replicates for RNA sequencing based on having aerial dry weights closest to the mean for that treatment’

11. **Results:L408. Somehow, fungal treatment can offset the negative effect of millet on plant growth?**

This is correct, and is elaborated on further in the paragraph.

12. **L414. Fig. 3a**

fixed

13. **L416-417. From Fig. 3b, it seems that uninoculated control also had slightly higher average seed mass than NVP64wt and NVP80cu.**

This is reported on Ln 423: “Unlike total seed mass, the average seed mass of the Uninoculated control was slightly higher than NVP80wt and NVP64cu, but not significantly different from the NoMillet control (**Fig. 3b**)”. This pertains to the average mass of each seed (total seed mass/number of seeds).

14. **L428. Plants?**

We removed ‘fungal’ so it now reads ‘treatments’, which refers to the inoculum used (NVP64+, NVP64-, NVP80+, NVP80-, control).

15. **L433. Please correct the format of ref.**

fixed

16. **L443-451. It is not clear where are these data from. Please cite figures or tables here.**

Analysis of variance analyses were used to determine which factors were significant for inclusion in the linear models. Following standard convention, non-significant factors based on ANOVAs are reported only in the text directly.

17. **L485. How did you get this? Try to use statistical analysis (e.g., PERMANOVA) to reveal it.**

Thank you for suggesting we test this. Initially, this was an observation that there was no clustering based on the first two principal components in 2-dimensional space. Applying a PERMANOVA test in R (code shown at the bottom of the rnaseq.R file) revealed there was no significant clustering based on either isogenic strain or cured/wild-type.

18. **L508-509. Please show the specific GOs here and site Table 2.**

We have included specific GO numbers here and have cited table 2.

19. **Discussion: L529. It is surprising that bacterial endosymbionts negatively impact the growth of Linnemannia. According to the previous study (Büttner et al., 2021), bacterial endosymbionts (*Ca. Mycoavidus* necroximicus) can protect Mortierella/ Linnemannia from the attack of micro-predators.**

The negative impact of endosymbionts on *Linnemannia* growth in pure culture was previously published by Uehling et al 2017 and Desiro et al. 2018. It was not assessed in the Büttner et al., 2021 paper.

20. **L540-544. This sentence is too long. Try to break it down into two sentences.**

This sentence has been rewritten. “While it is difficult to draw strong conclusions with so few replicates, fitness trade-offs in seed size and seed mass of plants growing in stressful environmental conditions compared to non-stressful conditions is an interesting topic for future work [[47,48]](https://paperpile.com/c/2N2aIr/QjEp+a6WH).”

21. **L555. This sentence is less connected to previous ones. Is Serendipita (Piriformospora) indica phylogenetically close to *L. elongate*?**

Thank you. The sentence has been better connected by adding this transition.

“However, although *Serendipita* and *Linnemannia* may both be found in plant roots, these fungi are phylogenetically distant, as *Serendipita* are Basidiomycetes while *Linnemannia* belong to Mucoromycota. Without further experiments to characterize nutrient exchange through these intracellular structures, it is not possible to ascribe structure with function [[55]](https://paperpile.com/c/RquxcV/KAGsf).”

22. **L637. Refs?**

Refs added

23. **L649. Although K, P, and Fe are available in PNM medium, there is a great demand for plant growth in the fungal treatment. This is probably the main reason for so many down-regulated genes related to nutrients.**

**We have revised this sentence Ln643:** “Considering the availability of potassium, phosphorus, and iron in the PNM growth medium, it is striking that so many genes related to deficiencies of these nutrients were down-regulated compared to the control plants. The fungal mycelial network may have increased the availability of these nutrients to plant roots, by serving as an extension of the root system.”

24. **L651. Correct the format of ref.**

This has been fixed
